# A systematic review and critical evaluation of immunohistochemical associations in hidradenitis suppurativa

**DOI:** 10.12688/f1000research.17268.2

**Published:** 2019-06-17

**Authors:** John W. Frew, Jason E. Hawkes, James G. Krueger

**Affiliations:** 1 Laboratory for Investigative Dermatology, Rockefeller University, New York, NY, 10065, USA

**Keywords:** Hidradenitis Suppurativa, Cytokeratin, Immunohistochemistry, Pathogenesis, Inflammation, Follicular Occlusion

## Abstract

**Background: **Hidradenitis suppurativa (HS) is a chronic inflammatory disease with significant morbidity and impact on quality of life. Our understanding of the pathophysiology is incomplete, impairing efforts to develop novel therapeutic targets. Immunohistochemistry studies have produced conflicting results and no systematic evaluation of study methods and results has been undertaken to date.

**Methods: **This systematic review aimed to collate and describe all reports of immunohistochemical staining in HS. This systematic review was registered with PROSPERO and conducted in line with the PRISMA reporting guidelines. Potential bias was assessed using the NIH Criteria and antibodies used across various studies were tabulated and compared.

**Results**: A total of 22 articles were identified describing results from 494 HS patients and 168 controls. 87 unique immunohistochemical targets were identified. The overall quality of studies was sub-optimal with staining intensity confounded by active treatment. Conflicting data was identified and able to be reconciled through critical evaluation of the study methodology.

**Conclusions**: Keratinocyte hyperplasia with loss of cytokeratin markers co-localizes with inflammation comprising of dendritic Cells, T-lymphocytes and macrophages, which are known to play central roles in inflammation in HS. Primary follicular occlusion as a pathogenic paradigm and the principal driver of HS is unclear based upon the findings of this review. Inflammation as a primary driver of disease with secondary hyperkeratosis and follicular occlusion is more consistent with the current published data.

## Introduction

Hidradenitis suppurativa (HS) is a chronic inflammatory disease, the exact pathophysiology of which remains incompletely defined ^1^. Numerous inflammatory mediators including TNF-α ^2^, IL-17 ^2, 3^, IL-32 ^4^ and IL-36 subtypes ^5, 6^ have been implicated in the disease. However, there is an incomplete understanding of the source and triggers of these mediators and how they sustain the chronic inflammation that characterizes this disease ^1, 2^. The pathogenic paradigm of HS has evolved dramatically since the first description by Velpau in 1839 ^7^. First thought of as an apocrinitis of infectious aetiology, it is now considered a disorder of follicular occlusion and more recently an inflammatory disease characterised by a keratinocyte mediated inflammatory response ^6^. However, the variable response to topical, systemic and biologic therapies in HS ^8^ indicate our understanding of disease pathophysiology is incomplete when compared to other cutaneous inflammatory diseases such as psoriasis ^9^ and atopic dermatitis ^10^. Existing studies examining the histology and immunohistochemical profiling of HS tissues represent conflicting results, for example in the degree of dermal dendritic cell infiltration ^11, 12^ and the production of TNF-alpha in the follicular unit ^13, 14^. These results may be influenced by heterogeneous sampling methods, laboratory processing methods and data analysis ^15^. An additional complicating factor is that clinical comorbidities which are strongly associated with disease activity in HS, such as obesity ^16^, diabetes ^17^, inflammatory bowel disease ^18^, and smoking ^19^ also impact inflammatory cell activity in the skin ^18, 20– 22^. Hence it remains unclear whether the presence or absence of these conditions may confound the findings of immunohistochemical studies in HS ^15, 23^ and whether clinical stratification of patients is required to identify distinct pathogenic pathways, which may be amenable to pharmacological intervention. This variability across studies makes comparing data problematic. To date no systematic analysis of immunohistochemical studies has been undertaken to compare results, methodology and analytical techniques.

## Objectives

The objectives of this systematic review are:

1) To collate and describe all published reports of immunohistochemical studies in HS
2) To critically evaluate the sampling, laboratory and analysis techniques used in each study to determine if comparisons can be made across studies.

## Methods

This systematic review was registered with PROSPERO ^24^ (Registration number CRD42018104763) and was conducted in line with the PRISMA ^25^. The STROBE statement ^26^ was used to assess the observational studies included in this study.

### Data sources

**Figure 1.**
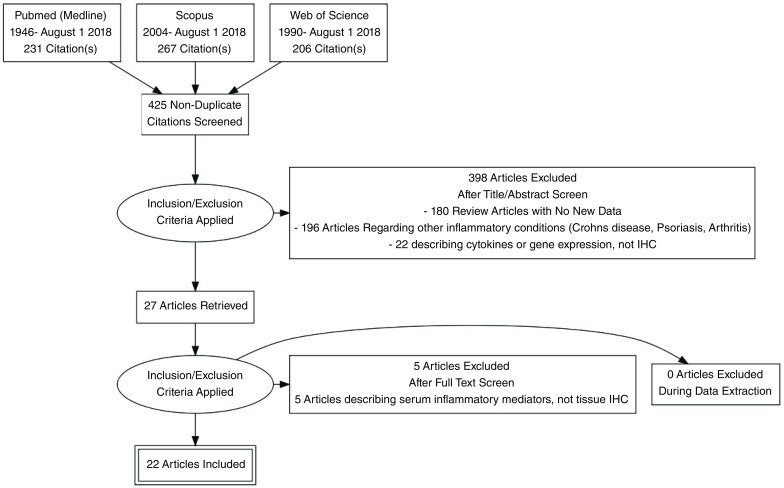
PRISMA flowchart.

Information Sources for this review encompassed Pubmed (1946-July 1 2018), Scopus (2004- July 1 2018) and Web of Science (1990-July 1 2018) as shown in Figure 1. Search strategy is presented as Table 1.

**Table 1.** Search Strategy for Systematic Review Entitled *“A Systematic Review and Critical Evaluation of Immunohistochemisty Studies in Hidradenitis Suppurativa*.

Resources:	
	1) Pubmed (1946-July 1 2018), 2) Scopus (2004- July 1 2018) 3) Web of Science (1990-July 1 2018) 4) Published Abstracts 5) Contact with Authors for abstracts without full text for clarification of data and methodology
Pubmed Search Strategy:	
	acne inversa OR apocrine acne OR apocrinitis OR Fox-den disease OR hidradenitis axillaris OR HS OR pyodermia sinifica fistulans OR Velpeau’s disease OR Verneuil’s disease OR Hidradenitidis Suppurative AND IHC OR Immunohistochemistry OR Histology

### Study eligibility criteria

Eligibility criteria for this review included cohort studies, case-control studies and other observational studies with no restrictions of patient age, sex, ethnicity or language of publication. Eligible studies included those reporting the results of immunohistochemical findings in HS. Studies deemed not eligible included articles which provided no new data, only a review or summary of previously published data.

### Appraisal and synthesis methods

Data collection was performed independently by 2 authors (JWF & JEH), with any disagreements regarding inclusion of citations being referred to a third author (JGK) for mediation. Information was collected using a standardized data collection form (available as Extended data ^27^) with the principal outcomes of interest being the immunohistochemical stain of interest, the site and rated intensity of staining (as described by authors), and comparison with perilesional/ unaffected/ control tissue. If data from individual patients was not available then the aggregate data was collected.

Potential sources of bias in the identified studies are acknowledged including the small size of patient cohorts, the variability in sampling and laboratory techniques, antibodies published and reactants used. Therefore these variables (where available) were collated to assess the heterogeneity of studies. Bias was also assessed using the NIH quality assessment tool for observational studies ^28^.

## Results

A total of 425 non-duplicated citations were identified in the literature review ( Figure 1). 398 of these articles were removed upon review of titles and abstracts against the pre-defined eligibility criteria. Full text review of the remaining 27 articles excluded 5 articles providing no new data. The remaining 22 studies ^4– 6, 11– 14, 29– 43^ reporting the results of 494 individual HS patients and 168 control patients were used as the basis of this systematic review.

### Descriptive analysis

The demographics of the patients of the included studies are presented in Table 2. Of 494 HS patients, 180 were male (38.3%) and 290 female (61.7%) with 24 cases unreported. Ages ranged from 15–72 years. 47/50 (94%) of reported cases were smokers, 12/30 (40%) had a BMI >30, and there was no information pertaining to diabetes or family history of HS. Of the 200 documented biopsy sites 93 were axillae (46.5%), 69 were inguinal (34.5%), and 38 were genital (19%) ( Table 3). 64 patients had Hurley staging with 7/64 (10.9%) Stage 1, 40/64 (62.5%) Stage 2 and 17/64 (26.6%) Stage 3. No individual Sartorius scores were reported. Where current treatment was reported, 6 patients (4.5%) were on adalimumab, 42 (31.6%) were untreated, 85 patients (63.9%) had treatment withheld prior to biopsy, and 357 cases were unreported. Lesional biopsies were taken from all studies, with 3 individual studies also taking perilesional biopsies ^6, 30, 41^. Age and Sex matched controls were present in 3 studies ^29, 31, 43^ and results were stratified in a minority of studies. 2 studies stratified by disease severity ^4, 12^, 7 studies stratified by lesion site ^13, 33– 37, 39^, 5 studies stratified by treatment ^4, 5, 12, 29, 32^, and no studies stratified by comorbidities. Analysis of immunohistochemical staining methodology varied and included quantitative analysis (3 studies) ^14, 30, 32^, semi-quantitative analysis (14 studies) ^4, 5, 11– 13, 29, 34, 37– 43^, and the presence or absence of staining (5 studies) ^6, 31, 33, 35, 36^. A total of 87 distinct immunohistochemical staining targets were identified ( Table 4, Table 5 and Table 6).

**Table 2.** Demographic data of included studies.

Number of HS Patients	Male	Female	Mean Age (Years)	Comorbidities				Biopsy Sites			Hurley Staging	mHSS Score (Mean)	Therapy	Study Reference
				Smoking	Obesity (BMI>30)	Diabetes	Family History	Axillae	Groin	Genital				
18	11	7	(Range 19–62)	NR	NR	NR	NR				NR	NR	NR	**14**
15	6	9	38.7	NR	NR	NR	NR	9	4	2	Stage 1=0 Stage 2=10 Stage 3=5	NR	Nil	**6**
24	8	16	36.5 (range 21–51)	NR	NR	NR	NR	NR	NR	NR	Mean=2.29 (SD=0.62)	NR	Untreated	**29**
22	10	12	38.2 (Range 19–60)	NR	NR	NR	NR	NR	NR	NR	NR	NR	NR	**30**
10	5	5	42 (Range 21–49)	NR	NR	NR	NR	Y	Y	N	Stage 2 (100%)	NR	Treatment Withheld	**32**
20	8	12	37.5 (Range 21–51)	N=18	N=10	NR	NR	NR	NR	NR	NR	NR	Treatment Withheld (8 weeks)	**4**
25	9	16	36 (Range 18–51)	NR	NR	NR	NR	NR	NR	NR	Mean =2.16 (SD=0.55)	NR	Treatment Withheld (3 weeks)	**5**
47	19	28	42.3 (Range 22–54)	NR				NR	NR	NR		48.3 (Range 8–144)	NR	**31**
11	9	2	39.6 (Range 18–61)	NR	NR	NR	NR	NR	NR	NR	“Mod-Severe Disease”	NR	NR	
20	6	14	40 (SD=15)	19	27.6 (4.1)	NR	NR	7	12	1	Stage 1=4 Stage 2=11 Stage 3=5	NR	Treatment withheld 3 weeks prior	**12**
10	1	9	38 (SD=15)	10	28.9 (SD 4.5)	NR	NR	3	7	0	Stage1=2 Stage2=7 Stage3=1	NR	Treatment Withheld 3 weeks prior	
14		1	30	NR	NR	NR	NR	1		0	NR	NR	NR	**13**
		1	42	NR	NR	NR	NR		1		NR	NR	NR	
		1	25	NR	NR	NR	NR		1		NR	NR	NR	
		1	22	NR	NR	NR	NR	1			NR	NR	NR	
		1	45	NR	NR	NR	NR		1		NR	NR	NR	
		1	27	NR	NR	NR	NR	1			NR	NR	NR	
	1		38	NR	NR	NR	NR	1			NR	NR	NR	
	1		34	NR	NR	NR	NR	1			NR	NR	NR	
		1	59	NR	NR	NR	NR		1		NR	NR	NR	
		1	41	NR	NR	NR	NR	1			NR	NR	NR	
		1	33	NR	NR	NR	NR	1			NR	NR	NR	
		1	46	NR	NR	NR	NR		1		NR	NR	NR	
		1	49	NR	NR	NR	NR		1		NR	NR	NR	
	1		31	NR	NR	NR	NR		1		NR	NR	NR	
60	26	34	37.3 (Range 15–67)	NR	NR	NR	NR	1	6	1	NR	NR	NR	**33**
9	1		47	NR	Y	NR	NR		1		3	NR	adalimumab	**11**
		1	31	NR	N	NR	NR		1		1	NR	adalimumab	
	1		24	NR	N	NR	NR	1			3	NR	adalimumab	
		1	32	NR	N	NR	NR			1	3	NR	adalimumab	
		1	58	NR	N	NR	NR			1	3	NR	adalimumab	
	1		58	NR	N	NR	NR			1	2	NR	adalimumab	
	1		36	NR	Y	NR	NR		1		2	NR	Nil	
	1		39	NR	N	NR	NR		1		3	NR	Nil	
	1		67	NR	N	NR	NR			1	3	NR	Nil	
16	1	15	NR	NR	N	NR	NR	3		13	NR	NR	NR	**34**
5	1	4	18-36	NR	NR	NR	NR	2	3		NR	NR	NR	**35**
50	18	32	11-70	NR	NR	NR	NR	39	6	5	NR	NR	NR	**36**
14	11	3	16-72	NR	NR	NR	NR	2	12	0	NR	NR	NR	**37**
15	NR	NR	NR	NR	NR	NR	NR	NR	NR	NR	NR	NR	NR	**38**
22	6	16	45.6 (Range 29–69)	NR	NR	NR	NR	13	7	2	NR	NR	NR	**39**
9	3	6	44 (Range 32–70)	NR	NR	NR	NR	NR	NR	NR	NR	NR	NR	**40**
12	0	12	29.4 (Range 19–42)	NR	NR	NR	NR	3	0	9	NR	NR	NR	**41**
36	13	23	25 (Range 20–69)	NR	NR	NR	NR	NR	NR	NR	NR	NR	NR	**42**
10	NR	NR	NR	NR	NR	NR	NR	NR	NR	NR	NR	NR	NR	**43**
**494**	**180**	**290**		**47/50** **Reported**	**12/30** **Reported**	**None** **Reported**	**None** **Reported**	**93/200**	**69/200**	**38/200**	**Hurley 1= 7** **Hurley 2= 40** **Hurley 3= 17** **Unknown= 430**	**No** **Individual** **Scores** **Reported**	**adalimumab=6,** **untreated=42,** **treatment** **withheld=- 85,** **not reported=** **357**	

BMI= Body Mass Index mHSS= modified Hidradenitis Suppurativa Score (Sartorius Score) NR= Not Reported

**Table 3.** Critical Evaluation of Methodology of Studies Included in This Review.

IHC Targets	Number of HS Patients	Number of Controls	Samples Analyzed	Age/Sex Matched Controls	Stratified by severity	Stratified by lesion site	Stratified by Co- morbidities	Stratified by Treatment	Immunostaining Intensity Assessment	Study Reference
α-MSH, LL37, S100A7, MIF, TNF-α, hBD3, lysozyme	18	12	L	N	NR	N	N	N	Quantitiative Immunohistomorphometry (Image J Software)	14
IL36	15	15	L, PL	NR	NR	N	N	N	Present/ Absent	6
CD3, CD56 LL37	24	9	L	Y	NR	NR	N	Y (untreated)	Semiquantitative (0-3)	29
CD1a, CD4, CD8 CD20, CD56, Factor XIIIa, IL17, NLRP3, Caspase-1	22	Yes (NR)	L, PL, U, C	NR	N	N	N	N	Cell Counting square grid x400 magnification	30
IL-23, IL-12, CD68, CD4	10	8	L, C	N	N	N	N	Y (ceased 3/52 prior)	Positive stained cells per mm ^2^	32
IL-32	20	10	L, C, S	N	Y	N	N	Y (ceased 8/52 prior)	Semiquantitative (+ to ++++)	4
IL-36	25	7	L, C, S	N	N	N	N	Y (ceased 3/25 prior)	Semiquantitative (+ to ++++)	5
LCN2	10	16	L	Y	N	N	N	N	Present/ Absent	31
CD11c	20	6	L	N	Y	N	N	Y	Semiquantitative (+ to ++++)	12
MMP2 hBD2 TNF-α	14	2	L, C	N	N	Y	N	N	Semiquantitative ((+ to ++++)	13
CD3, CD4, CD8, CD68 CD79 CD56	60	Yes (NR)	L,C	N	N	Y	N	N	Present or Absent	33
CD3, CD4, CD8, CD20, CD138, CD14, CD68, CD11c	9	Yes (NR)	L,C	N	N	N	N	N	Semiquantitative (+ to ++++)	11
GCDFP-15, CD15, Lysosyme, S100, Ca19-9, HMB45	13	3	L,C	N	N	Y	N	N	Semiquantitative (+ to ++++)	34
CD29, CTx-FITC	5	4	L,C	N	N	Y	N	N	Present or Absent	35
AE1/AE3/PKC26/ Enhanced Alkaline Phosphatase	50	Y (NR)	L,C	N	N	Y	N	N	Present or Absent	36
K1, K10, K14, K16,k17, K19,	14	1	L,C	N	N	Y	N	N	Semiquantitative (+ to ++++)	37
Desmoplakin 1,2, Plakoglobin, Plakophilin 1,2, Desmoglein 1,2,3, Desmocollin 1,2,3,K2e, K4, K5, K6, K7, CK8,CK9, CK10, K13, K13/15/16, K14, K17,K19, K20 Ki67	15	Y (NR)	L,C	N	N	N	N	N	Semiquantitative (+ to ++++)	38
ER, AR	22	10	L,C	N	N	Y	N	N	Semiquantitative (+ to ++++)	39
TLR2, CD3, CD19, CD56,CD68, CD11c, CD1a, CD206, CD207, CD209	9	Y (NR)	L,C	N	N	N	N	N	Semiquantitative (+ to ++++)	40
TLR 2,3,4,7,9,ICAM-1, TNF-α, IL-6, IL-10, TGF-β, α-MSH, hBD2, hBD4 IGF-1	12	Y (NR)	L,PL	N	N	N	N	N	Semiquantitative (+ to ++++) Epidermis Only	41
hBD3, S100A7, RNase7	36	57	L,C	N	N	N	N	N	Semiquantitative (+ to ++++)	42
MMP8	10	8	L,C	Y	N	N	N	N	Semiquantitative (+ to ++++)	43
	494	168		**3/22**	**2/22**	**7/22**	**0/22**	**5/22**		

Table 3: Critical Evaluation of Methodology of Studies Included in This Review Key:L= Lesional, PL= Perilesional, U= Uninvolved, C= Control S=Serum, Y=Yes, N=No, NR= Not Reported, CTx-FITC =Cholera Toxin

**Table 4.** Data pertaining to distribution of cells expressing Immunohistochemical markers described in this review.

Cell Type	Study	Results
Basal Keratinocytes	13	MMP2 Expressed
Suprabasal Keratinocytes	31	LCN 2 staining of suprabasal keratinocuytes
Dermal Fibroblasts	13	MMP2 expressed
	7	33/51 specimens associated with ++ fibrosis
Neutrophils	13	MMP2 in keratinocytes, fibroblasts, macrophages and lymphocytes,
	30	Significant increase in number of neutrophils in dermis Dermis> Perifollicular
	31	LCN2 in neutrophils – epidermis and dermis
	7	+++ infiltrate in 29/51 specimens
Plasma Cells	7	+++ Plasma cell infiltrate in 2/51 specimens
Eosinophils	7	+++ Eosinophilic infiltrate ++ in 3/51 specimens
Histiocytes	7	+++ Infiltrate 24/51 speciments
Lymphocytes (NOS)	13	MMP2 expressed TNF alpha positivity in dermis
	44	Lymphocytes, giant cell and necrosis in established lesions
T Cells	4	CD3 + Dermis producing IL32C D 56 + NK T cells producing IL32
	33	lymphocytic mixed infiltrate perifollicular (with unruptured terminal follicles). This consisted of CD-3 (39%), CD-4 (30%), CD-8 (14%), and positive cells (CD-4 ⁄ CD-8 ratio: 2.1:1). CD-56 (0.1%) and UCHL-1 (0%) brought no conclusive results. Conspicuous was a CD-8 cell positive folliculotropism in all immuno- histologies (Figure 3). CD-8 positive lymphocytes were loosely distributed not only in the stratum basale but also in the suprabasal epithelial areas. The subepidermal inflammatory infiltrate in the area of interfollicular epidermal hyperplasia showed a comparable cellular composition: CD-3 (38%), CD-4 (26%), CD-8 (19%), CD-56 (0.2%) and UCHL-1 (0%), CD-4 ⁄ CD-8 Ratio: 1.4:1. Here too, a CD-8 positive pronounced epidermotropism was impressive
	30	At perifollicular sites, quantitative analysis showed a significant increase in the mean number of CD3+, CD4+ and CD8+ T lymphocytes (CD3+, 34 ` 20 per HPF; CD4+, 38 ` 21; CD8+, 12 ` 8) compared with healthy control skin (CD3+, 9`4; CD4+, 2`1; CD8+, 1`1;
	3	CD4 T cells producing IL17 in dermis
	32	CD4 T cells producing IL17 in dermis
B Cells	11, 12	Psuedolymphomatous nests (see cytokine studies)
	33	Perifollicular infiltrate with unruptured terminal follicles: CD-79 (35%) Subepidermal interfollicular Infiltrate: CD-79 (33%),
Dendritic Cells	11	Successful Adalimumab treatment reduced influx of CD11c+ dendritic cells in lesional skin
	12	Number of dendritic cells stable in skin- mild elevation only
	4	Dermis producing IL32
Macrophages	13	MMP2 expressed TNF alpha positivity
	30	Significant increase in deep infiltrate
	32	Increase with co-staining of CD68/CD32 and IL12/ IL23
	33	Perifollicular infiltrate with unruptured terminal follicles: CD-68 (12%) Subepidermal interfollicular Infiltrate: CD-68 (19%),
	4	Dermis producing IL32
Mast Cells	30	Significant increase in deep infiltrate
	12	Significant increase in deep infiltrate

**Table 5.** Reported Immunohistochemical Staining Results Identified in this Systematic Review.

IHC Staining Target	Epidermis		Dermis	Hair Follicles		Sinus Tracts				Subcutis	Apocrine/ Eccrine Glands	Study Reference
	Suprabasal Staining	Basal Staining	Dermal Staining	Infundibular Staining	ORS Staining	Type 1		Type 2	Type 3			
						Type A	Type B	Type C				
CD1a			+									30
	++		+									40
			+++									12
CD3	++		++		++							30
	+	+	+									40
												33
CD4			++									12
			Interfollicular and perifollicular									33
			+		+							30
												32
CD8			+									12
	Epidermotropism		Interfollicular and perifollicular									33
			+		+							30
CD11c	+++											12
			+++									40
CD14												12
CD15											+	34
CD19	-		+									40
CD20			+++									30
			+++									12
CD29	+	+		+		+ (NOS)						35
CD32												32
CD56												30
	-		+									40
CD68	Deep> Perifollicular		++		+							30
			Interfollicular and perifollicular									33
	+		+++									40
												32
CD79			Interfollicular and perifollicular									33
CD138			Mild infiltrate									12
CD206	+		+++									40
CD207	+++ *		+									40
CD209	++		++++									40
Cytokeratins												
AE1			Single K									36
AE3			Single K									36
PKC26			Single K									36
Factor XIIIa			DC +									12
			+		+							30
K1	Present in acanthotic epidermis			+	-	+	+	-			-	37
K2e	++					-		-	-			38
K4	-					-		-	-			38
K5	+					+		+	+			38
K6	-					+		+	+			38
K5/6 **^§^**	++++											30
K7	-					-		-	+			7
K8	-					-		-	-			38
K9	-					-		-	-			38
K10											-	36
	Present in acanthotic Epidermis			+	-	+	+	-			-	37
	++	+				++		-	-			38
K13	-					+		+	-			38
K13+15+16 **^§^**	+	+				+		+	+			38
K14	Highly positive in acanthotic epidermis			+	+	+	+	++			Sebaceous Duct and Gland +	37
						+		+	+			38
K15											Stained apocrine glands	34
K16	Weakly positive in acanthotic epidermis			-	+	-	+	+			-	37
K17	Weakly positive in acanthotic epidermis			-	+	-	+	+			-	37
K18												38
K19						+ NOS					+	36
	Weakly positive in acanthotic epidermis			-	+	-	-	-			-	37
						-		-	++			38
K20						-		-	-			38
Ki67	+					++		++	++			38
ER	-										+	39
AR	-										NC	39
GCDFP-15											Apocrine glands	34
S100											Eccrine glands	34
Lysozyme											Vulval cases only	34
		↓ in scarred cases										14
HMB45	-										Negative all cases	34
TLR2	++		++++									40
	↓											41
TLR3	↓											41
TLR4	↓											41
TLR7	↓											41
TLR9	↓											41
ICAM-1	↓											41
TGF-β	↓											41
IGF-1	↓											41
RNase7	+++											42
MMP2	+++/++++	+++/++++	+		+++	+ NOS						13
MMP8	(Neutrophils)		+++			(Neutrophils)						43
Cholera Toxin	Slopes of papillae suprabasal epidermis,			hair follicles		+ NOS						35
Desmoplakin 1	++					++		++	+			38
Desmoplakin 2	++					++		++	+			38
Plakoglobin	++					++		++	+			38
Plakophilin 1	++					++		++	+			38
Plakophilin 2						-		-	-			38
Desmoglein 1	++	+				++		++	-			38
Desmoglein 2	+					+		++	++			38
Desmoglein 3	++					++		++	+			38
Desmocollin 1	++	+				++		-	-			38
Desmocollin 2	++	+				++		++	+			38
Desmocollin 3	++					++		++	+			38
hBD2	↓										Negative in 12/14	1
	↓											41
hBD3	++ (suprabasal)	-			++							14
	+++	+										42
hBD4	↓											41
TNF- **α**	++/+++ (macrophage/ lymphocytes)				++/+++						+++	13
	++	++	+	NC	↓							14
IL-6	↓											41
IL-10	↓											41
IL-12			++++									32
IL-23			++++									32
IL-17			Diffuse									30
			+++									32
IL-32		++	+++									4
IL-36	+	+++										6
	Suprabasal	+++										5
Caspase1	++											30
NLRP3	++											30
MIF	++				++							14
S100A7	++	+++			++							14
	++											41
LL-37	++				++						NC	14
		++	+++		++							29
**α** -MSH	++				NC							14

Key: + to ++++ = Degree of positive staining, - = reported negative staining, NC= No Change; ↓ Decreased, NOS= Not Otherwise Specified, *= Statistically significant result compared with healthy controls, §=Pan Cytokeratin Stain, DC= Dendritic Cells, Single K= Single keratinocytes,

**Table 6.** Immunohistochemistry stains/antibodies used in included reviews.

Target	Details	Study Reference
CD1a	CloneO10; Dako Cytomartion	30
	CloneO10; Dako Cytomartion	40
	O10 1:20 Immmunotech, Prague, Czech Republic	12
CD3	clone F7.2.38; Dako)	30
	Polyclonal rabbit anti-human CD-3 dilution 1:25; Dako Cytomation Denmark A ⁄ S, Glostrup, Denmark),	33
	Polyclonal 1:150 Dako, Glostrup, Denmark	12
	Clone PC3/188A; DakoCytomation, Glostrup, Denmark	40
CD4	4B12 1:160 Monosan Uden The Netherlands	12
	monoclonal mouse anti-human CD-4 dilution 1:10; Vision Biosystems Novocastra, Newcastle, UK	33
	clone 4B12; Dako	30
	MT310 Dako	32
CD8	C9/144B 1:100 Dako	12
	monoclonal mouse anti-human CD-8 dilution 1:50; Dako Cytomation Denmark A ⁄ S	33
	clone C8/144B; Dako	30
CD11c	5D11 1:60 Novocastra Newcastle Upon Tyne, UK	12
	Clone KB90 DakoCytomation	40
CD14	MY4 1:100 Novocastra Newcastle Upon Tyne, UK	12
CD15	Not Reported	34
CD19	Clone HD37; DakoCytomation	40
CD20	clone L26 (1,4); Dako	30
	L 26 1:400 Dako	12
CD29	fluorescein-tagged B-subunit of cholera toxin (CTx-FITC) + CyChrome (Pharmingen BD Biosciences, Franklin Lakes, NJ, USA)	35
CD32	KB61 Dako	32
CD56	clone 123C3; Dako	30
	Clone MOC-1; DakoCyotmation	40
	monoclonal mouse anti-human CD-56 1:50; Dako Cytomation Denmark A ⁄ S),	33
	123C3.D5 1:25 Thermo Fisher Scientific Altrincham UK	12
CD68	Clone PG- M1; Dako)	30
	monoclonal mouse anti-human CD-68 dilution 1:50; Dako Cytomation Denmark A ⁄ S)	33
	Clone EBM11; Dako Cytomation	40
	KP1 1:160 Dako	12
	EBM11 Dako	32
CD79a	monoclonal mouse anti-human CD-79 dilution 1:25; Dako Cytomation Denmark A ⁄ S)	33
	JCB117 1:100 Dako	12
CD138	B-A38 1:25 IQ Products Groningen, The Netherlands	12
CD206	Clone 19.2; BD Biosciences Pharmingen	40
CD207	Clone DCGM4; Immunotech, Marseilles, France	40
CD209	Clone DCN46; BD Biosciences Pharmingen, San Diego Ca USA	40
Cytokeratins		
Pankeratin	AE1/AE3/PKC26; Ventana Medical Systems SA, Illkirch, Cedex, France	36
	AE1/AE3 1:200 Thermo Fisher Scientific	12
Factor XIIIa	AC-1A1 1:200 Thermo Fisher Scientific	12
	clone E980.1; Leica Biosystems Newcastle, Newcastle upon Tyne, U.K.)	30
K1	34 Beta B4 Novo Castra Laboratories Ltd, Newcastle- upon-Tyne, UK)	37
K2e	Ks2́ 342́ 7.1 against CK 2e (Dr L.Langbein, Heidelberg, Germany),	38
K4	6B10 against CK 4,	38
K5	AE 14 against CK 5,	38
K6	Ks6.KA12 against CK 6,	38
K5/6	clone M7237; Dako	30
CK7	OV-TL 12/30 and Ks7 ´18 against CK 7,	38
K8	CAM 5 ´2 against CK 8,	38
K9	HK9TY1 (guinea-pig polyclonal) against CK 9 (Dr L.Langbein)	38
K10	Not Reported	36
	LHP1 Novo Castra Laboratories Ltd, Newcastle- upon-Tyne, UK)	37
	MoAbs K8́ 60 and DE-K10 against CK 10,	38
K13	Ks13 ´1 against CK 13,	38
K13+15+16	Ks8 ´12 against CK 13 15 16,	38
K14	LL001 Novo Castra Laboratories Ltd, Newcastle- upon-Tyne, UK)	37
	LL001 against CK 14,	38
K15	Not Reported	34
K16	LL025 Novo Castra Laboratories Ltd, Newcastle- upon-Tyne, UK)	37
K17	E3 Novo Castra Laboratories Ltd, Newcastle- upon-Tyne, UK)	37
	Ks17.E3 against CK 17,	38
K19	Not reported	36
	B170 Novo Castra Laboratories Ltd, Newcastle- upon-Tyne, UK)	37
	Ks19́1 against CK 19,	38
K20	IT-Ks20́10 against CK 20	38
Ki67	MIB 1 against Ki-67	38
	MIB1 1:100 Dako	12
ER	ER (Thermo Scientific; pretreatment EDTA, pH 9.0, dilution 1:80).	39
AR	AR (Santa Cruz; pretreatment citrate, pH 6.0, dilution 1:100)	39
GCDFP-15	Not Reported	34
S100	Not Reported	34
Lysozyme	Not Reported	34
	A0099 pAbG 1:100 rabbit antihuman Dako Corporation	14
HMB45	Not Reported	34
TLR2	Clone TL2.3; Alexis Corp. San Diego Ca USA	40
	Santa Cruz Biotechnology, Inc, Santa Cruz, California	41
TLR3	Santa Cruz Biotechnology, Inc, Santa Cruz, California	41
TLR4	Santa Cruz Biotechnology, Inc, Santa Cruz, California	41
TLR7	Santa Cruz Biotechnology, Inc, Santa Cruz, California	41
TLR9	Santa Cruz Biotechnology, Inc, Santa Cruz, California	41
ICAM-1	Beckman Coulter, Inc, Brea, California	41
TGF-β	AbD Serotec	41
IGF-1	R&D Systems, Inc, Lille, France	41
RNase7	Dako	42
MMP2	MMP-2 (cat no. AF902, LOT DUB034081, obtained from goat, 1:100 dilution, R&D Systems	13
MMP8	Dako	43
Cholera Toxin	fluorescein-tagged B-subunit of cholera toxin (CTx-FITC) + CyChrome (Pharmingen BD Biosciences, Franklin Lakes, NJ, USA)	35
Desmoplakin 1	DP 1 2±2́15 and DP 1±2́17 against DP I II,	38
Desmoplakin 2	DP 1 2±2́15 and DP 1±2́17 against DP I II,	38
Plakoglobin	PG 5́1 and PG 11E4 (Dr M.J.Wheelock, Toledo, OH, U.S.A.) against PG,	38
Plakophilin 1	PP1-9E7 and PP1-5C2 against PP 1,	38
Plakophilin 2	PP2- 150 against PP 2,	38
Desmoglein 1	Dsg1E-P124 and Dsg1E-P23 against Dsg1,	38
Desmoglein 2	Dsg2E-G129 and Dsg2E- G96 against Dsg2,	38
Desmoglein 3	Dsg3-G194 and 5G11 against Dsg3,	38
Desmocollin 1	Dsc1-U100 against Dsc1,	38
Desmocollin 2	DC-Rab 36 (rabbit polyclonal) against Dsc2,	38
Desmocollin 3	MoAb Dsc3-U114 against Dsc3,	38
hBD2	Human beta-defensin 2 (cat no. AF 2758, LOT VJU015051, obtained from goat, 1:100 dilution, R&D Systems, Germany)	13
	Abcam, San Francisco, California	41
hBD3	1:400 rabbit antihuman Donated by Prof Schroders Labor Kiel germany	14
	1:1000 rabbit ani-human PeproTech, Rocky Hill, N J	42
hBD4	Abcam, San Francisco, California	41
TNF-α	TNF-α (code ab 6671, obtained from rabbit, 1:100 dilution, Abcam, Cambridge, UK	13
	559071 mAbG 1:10 mouse antihuman R&D Systems	14
IL-6	AbD Serotec, Oxford, England	41
IL-10	R&D Systems, Inc, Minneapolis, Minnesota	41
IL-12	IL-12p7024945 R&D Systems	32
IL-23	IL23p19 HLT2736 Biolegend	32
IL-17	clone AF-317-NA; R&D Systems, Wiesbaden Germany	30
	Polyclonal R& D Systems	32
IL-32	NBP-76684, Novus (Littleton, CO, U.S.A.)	4
IL-36	rabbit polyclonal anti-IL-36a (C-terminal; ab180909), rabbit polyclonal anti- IL-36b (C-terminal; ab180890) and mouse monoclonal anti-IL-36c (ab156783; all from Abcam, Cambridge, U.K.	6
	AF 1078,1099,2320,1275 RnD	5
Caspase1	clone 14F468; Imgenex/Novus Biologicals, Littleton, CO, U.S.A.)	30
NLRP3	clone Ab17267; Abcam, Cambridge, U.K.	30
MIF	MAB289 mAbG 1:100 mouse antihuman	14
S100A7	HL15-4 mAbG 1: 20,000 mouse antihuman Donated by Prof Schroders Labor Kiel germany	14
LL37/ Cathelicidin	Ab64892 pAbG 1:1000 rabbit antihuman Abcam	14
	Rabbit anti-human LL-37 [Abcam, Cambridge, UK	29
α-MSH	M0939 1:500 Rabbit Anithuman Sigma	14
	PROGEN Biotechnik GmbH, Heidelberg, Germany	41
Tryptase	AA1 1:800 Dako	12
	clone AA1; Dako	30

### Immunohistochemistry results

***Epidermis.*** The epidermis of HS lesional tissue expressed the normal array of keratins (K) in the basal (K5, K14) and suprabasal (K1, K2e, K10) layers. K6, K16 and K17 staining were increased compared to healthy controls in the suprabasal epidermis in one study ^30^, however, K6 and K17 staining was not increased in the epidermis (only in non-keratinized portions of sinus tracts) in a second study ^38^. Where K6 and K17 were positive in suprabasal epidermis, K17 staining was more pronounced than K6 staining ^30^. K19 was weakly positive in acanthotic epidermis ^37^. Ki67 staining was elevated in basal and suprabasal epidermis. Normal staining patterns of desmoplakin, plakophilin and plakoglobin were seen ^38^. Cells staining positive for CD1a, CD206, CD207 and CD209 were seen throughout the epidermis ^40^. CD3, CD4, CD8 and to a lesser degree CD68 positive cells demonstrated epidermotropism in sites of epidermal acanthosis ^30, 33^. CD29 and cholera toxin (double positive) staining cells were seen on the slopes of papillae of the epidermis ^35^. hBD2 (human beta defensin) staining was decreased throughout the epidermis in two studies ^13, 41^ whilst hBD3 staining was increased throughout the suprabasal epidermis ^14, 42^, however only significantly in Hurley Stage 1 and 2 patients (p=0.045) ^42^. hBD4 was decreased in suprabasal epidermis compared to healthy controls (p=0.001) ^41^. Contradictory findings were seen in toll like receptor (TLR) 2 staining with an increase in the epidermis co-localizing with dendritic cells and macrophages in one study ^40^ but suppressed in a second study ^41^. Levels of TLR3, TLR4, TLR7, TLR9, ICAM-1, TGF-Beta and IGF-1 were only assessed by one study and all were suppressed throughout the epidermis compared with controls ^41^. RNAase7 was increased in expression compared to healthy controls (p<0.05) ^42^. MMP2 was positively expressed in keratinocytes throughout the epidermis ^13^ and MMP8 in neutrophils within the epidermis ^43^. TNF-α was highly expressed in macrophages and lymphocytes present in the epidermis, particular in the basal layers ^13, 14^ and NLRP3, MIF, S100A7, LL37/Cathelicidin and α-MSH all positive in suprabasal keratinocytes ^30, 41^. IL-6 and IL-10 were reported as suppressed compared to healthy control skin ^41^, however, IL-36 subtypes were highly expressed in epidermal keratinocytes (more suprabasal than basal) ^5, 6^ with IL-32 also positive in the stratum granulosum ^4^.

***Dermis.*** CD1a, CD11c, CD206, CD207, CD209 and Factor XIIIa positive cells were identified in the dermis in three separate studies ^12, 30, 40^, however the degree of infiltration varied. Dermal infiltrates of CD3, CD4, and CD8 positive cells, continuous with the epidermal infiltrates were a consistent feature of lesional HS dermis and were increased over controls ^30, 33^. The distribution of these cells was most pronounced in the interfollicular dermis (ie. towards the papillary slopes) and perifollicularly (ie. peri-infundibularly) ^30, 33^. CD56, CD68 and CD138 positive cells were diffusely seen throughout the dermis ^40^. CD19 and CD20 positive pseudolymphoid follicles have been noted in other studies ^30, 40^. Single keratinocytes have also been identified in the dermis which stain with pancytokeratin markers (AE1/AE3/PKC26) ^36^. Inflammatory cells in the dermis co-localized with TNF-α ^13, 14^, LL-37/cathelicidin ^29^, IL-12 ^32^, IL-23 ^32^, IL-17 ^30, 32^, IL-32 ^4^, TLR2 ^40^ and MMP8 ^43^. MMP2 co-localized with macrophages and fibroblasts ^13^. IL-36 was not identified in the dermis ^5, 6^.

***Hair follicle.*** Cytokeratin staining of the follicular apparatus is consistent with normal K14, K16 and K17 staining. CD29 positive cells were identified in the infundibulum ^35^. CD3, CD4, CD8, CD68, Factor XIIIa positive cells were seen within the outer root sheath (ORS) contiguous with dense peri-follicular inflammation in the adjacent dermis ^30, 33^. The presence of inflammatory cells co-localized with MMP2 ^13^, TNF- α ^13, 14^, and LL37/cathelcidicin ^13, 29^. hBD3 ^13, 42^ and MIF ^13^ also stained positive in the ORS. One conflicting study reported no change in TNF-α staining of the follicular unit ^13^.

***Sinus tracts.*** Staining patterns differed between superficial keratinized sinus tracts and deeper, inflamed non-keratinized sinus tracts. Normal epidermal cytokeratin staining was seen in the keratinized superficial portion of sinus tracts including K1, K10, K14 ^36– 38^. Ki67 was elevated and CD29 positive cells were also identified in sinus tracts ^35^. Ki67 stained in both keratinized and non-keratinised portions of the sinus tract ^38^. K19 staining was absent in keratinized portions of sinus tracts ^37^. In deeper, inflamed, non-keratinized portions of the sinus tracts, K16, K17 and K19 were positive, with loss of K1, K10 and adhesions molecules including DG1 (desmoglein 1)and DSC1 (desmocollin 1) ^37, 38^. Apocrine gland nuclei stained weakly positive for estrogen receptor ^39^ and androgen receptor ^39^, and these results were reported as no different from control specimens ^39^. Lysozyme staining of apocrine glands was seen in cases of vulval HS only ^34^.

***Immunohistochemistry methods.*** The list of antibodies used for IHC staining is presented in Table 6. Consistent antibodies were used for CD1a; CD20 and tryptase staining, whilst different antibodies were used for other staining targets. Antibodies used were not described in two studies ^34, 36^.

***Assessment of Bias.*** The result of bias assessment using NIH criteria is presented in Table 7. All 22 articles clearly stated the research question of interest with well-defined study populations. The application of inclusion and exclusion criteria, or the calculation of sample size, or effect estimates were not described in any study. Exposures (ie. the presence of disease) were established and measured in all studies prior to the outcome measures (IHC staining) being assessed and the disease was established for such a time that a relationship between exposure and outcome would be identified if one existed. Different levels of exposure (severity of disease) was taken into account in only two studies ^4, 12^ and was consistently measured using Hurley staging across all studies. No articles accounted for all possible confounding variables such as obesity, diabetes, family history or smoking status ( Table 3).

**Table 7.** NIH Risk of Bias.

Study Reference	1. Was the research question or objective in this paper clearly stated?	2. Was the study population clearly specified and defined?	3. Was the participation rate of eligible persons at least 50%?	4. Were all the subjects selected or recruited from the same or similar populations (including the same time period)? Were inclusion and exclusion criteria for being in the study prespecified and applied uniformly to all participants?	5. Was a sample size justification, power description, or variance and effect estimates provided?	6. For the analyses in this paper, were the exposure(s) of interest measured prior to the outcome(s) being measured?	7. Was the timeframe sufficient so that one could reasonably expect to see an association between exposure and outcome if it existed?	8. For exposures that can vary in amount or level, did the study examine different levels of the exposure as related to the outcome (e.g., categories of exposure, or exposure measured as continuous variable)?	9. Were the exposure measures (independent variables) clearly defined, valid, reliable, and implemented consistently across all study participants?	10. Was the exposure(s) assessed more than once over time?	11. Were the outcome measures (dependent variables) clearly defined, valid, reliable, and implemented consistently across all study participants?	12. Were the outcome assessors blinded to the exposure status of participants?	13. Was loss to follow- up after baseline 20% or less?	14. Were key potential confounding variables measured and adjusted statistically for their impact on the relationship between exposure(s) and outcome(s)?
Emelianov *et al*. ^14^	Y	Y	N/A	N	N	Y	Y	N	Y	N	Y	NR	N/A	N
Hessam *et al*. ^6^	Y	Y	N/A	N	N	Y	Y	N	Y	N	Y	NR	N/A	N
Thomi *et al*. ^29^	Y	Y	N/A	N	N	Y	Y	N	Y	N	Y	NR	N/A	N
Lima *et al*. ^30^	Y	Y	N/A	N	N	Y	Y	N	Y	N	Y	NR	N/A	N
Schlapbach *et al*. ^32^	Y	Y	N/A	N	N	Y	Y	N	Y	N	Y	NR	N/A	N
Thomi *et al*. ^4^	Y	Y	N/A	N	N	Y	Y	Y	Y	N	Y	NR	N/A	N
Thomi *et al*. ^5^	Y	Y	N/A	N	N	Y	Y	N	Y	N	Y	NR	N/A	N
Wolk *et al*. ^31^	Y	Y	N/A	N	N	Y	Y	N	Y	N	Y	NR	N/A	N
Van der Zee *et al*. ^12^	Y	Y	N/A	N	N	Y	Y	Y	Y	Y	Y	NR	N/A	N
Mozeika *et al*. ^13^	Y	Y	N/A	N	N	Y	Y	N	Y	N	Y	NR	N/A	N
Von Laffert *et al*. ^33^	Y	Y	N/A	N	N	Y	Y	N	Y	N	Y	NR	N/A	N
Van der Zee *et al*. ^11^	Y	Y	N/A	N	N	Y	Y	N	Y	N	Y	NR	N/A	N
Heller *et al*. ^34^	Y	Y	N/A	N	N	Y	Y	N	Y	N	Y	NR	N/A	N
Gniadecki *et al*. ^35^	Y	Y	N/A	N	N	Y	Y	N	Y	N	Y	NR	N/A	N
Fismen *et al*. ^36^	Y	Y	N/A	N	N	Y	Y	N	Y	N	Y	NR	N/A	N
Kurokawa *et al*. ^37^	Y	Y	N/A	N	N	Y	Y	N	Y	N	Y	NR	N/A	N
Kurzen *et al*. ^38^	Y	Y	N/A	N	N	Y	Y	N	Y	N	Y	NR	N/A	N
Buiner *et al*. ^39^	Y	Y	N/A	N	N	Y	Y	N	Y	N	Y	NR	N/A	N
Hunger *et al*. ^40^	Y	Y	N/A	N	N	Y	Y	N	Y	N	Y	NR	N/A	N
Derno *et al*. ^41^	Y	Y	N/A	N	N	Y	Y	N	Y	N	Y	NR	N/A	N
Hofmann *et al*. ^42^	Y	Y	N/A	N	N	Y	Y	N	Y	N	Y	NR	N/A	N
Tsaousi *et al*. ^43^	Y	Y	N/A	N	N	Y	Y	N	Y	N	Y	NR	N/A	N
**Total**	**22/22**	**22/22**	**N/A**	**0/22**	**0/22**	**22/22**	**22/22**	**2/22**	**22/22**	**1/22**	**22/22**	**NR**	**N/A**	**0/22**

Key: Y = Yes; N= No, NR= Not Reported N/A = Not Applicable

## Discussion

### Quality of data and risk of bias

The overall quality of data in this systematic review was sub-optimal with poor correction for potential confounding factors with only two of the 22 studies using objective measurement systems for IHC staining intensity ^4, 12^. The proportion of smokers was elevated (94%) compared to the rates of smoking in the HS population at large (70–89%) ^45^. A number of studies (17/22) did not stratify results by treatment therefore there is a risk that staining intensity of pro-inflammatory mediators may be reduced due to concomitant treatment at the time of biopsy. The use of de-paraffinized tissue in retrospective studies ^30, 33, 34^ can lead to false negatives in IHC dependent upon the preparation method of the original sample and the de-paraffinization process ^15^. Hence there are factors in the population studied in this review which may bring into question the reliability of staining quantification. However, the presence or absence of IHC staining, particularly when confirmed in multiple studies is still considered reliable despite the risks of bias.

### Conflicting results

Conflicting results were identified in dermal CD1a staining ^12, 30, 40^, dermal CK19 staining ^36– 38^, Epidermal TLR2 staining ^40, 41^ and TNF alpha staining in the follicular infundibulum ^13, 14^. Regarding CD1a staining, two of the studies reported only a mild dermal infiltrate of CD1a positive cells ^30, 40^, with a third study demonstrating a significant infiltration of these cells ^12^. This third study clearly documented all treatment was withheld 3 weeks prior to the biopsies being taken ^12^, whereas there is no description in the other two articles regarding the discontinuation or ongoing use of treatments ^30, 40^. Therefore, with the possibility of partially treated disease, an artificial reduction in the number of dermal dendritic cells is a possibility as treatment for HS (such as adalimumab) has been demonstrated to effectively reduce the infiltration of dendritic cells *in vivo* ^12^. Similarly, studies examining TNF-alpha staining also differed in their stratification of patient based upon active treatment ^13, 14^. Significant reductions in TNF alpha staining were seen in the study with no documentation of treatment cessation ^14^ when compared to the one study with clear documentation that all patients had treatment ceased prior to biopsy ^13^. K19 staining was reported negative in all areas of the sinus tracts in one study ^37^, whereas two additional studies ^36, 38^ described positive K19 staining in sinus tracts (one study non-specifically ^36^ and the second in the deep inflamed, non-keratinized epithelium of the tract ^38^). The difference between these staining patterns may be explained by the presence of inflammation. Kurzen *et al*. ^38^ described the presence of K19 staining in non-keratinized epithelium of the deep sinus tracts only when associated with inflammation (Type 3 epithelia), staining was negative when no inflammation was present (Type 2 epithelia) ^38^. Kurokawa *et al*. did not differentiate between inflamed and non-inflamed non-keratinized epithelium in their study ^37^, and noted that the lesser degree of inflammation seen histologically may explain their differing results in comparison to Kurzen’s study ^37^.

### Localization of production of inflammatory mediators

**Figure 2.**
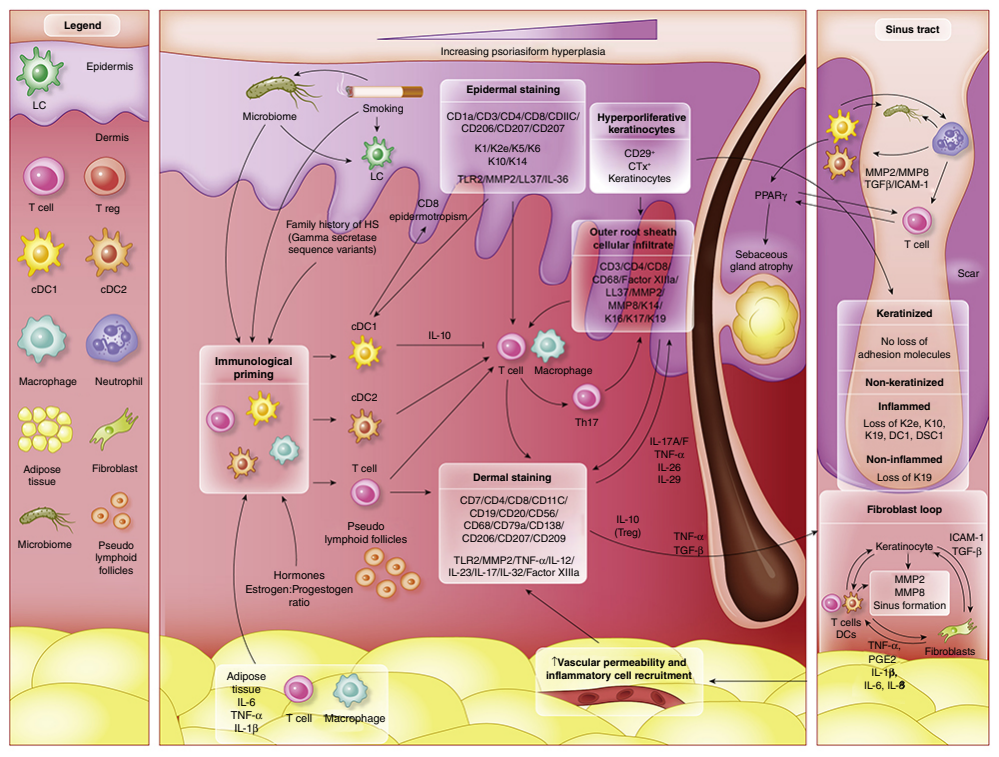
Schematic Representation of Immunohistochemical findings in hidradenitis suppurativa. Immunological ‘priming’ occurs due to the contribution of adipose tissue, genetic susceptibility, smoking-related inflammatory mediators and obesity related pro-inflammatory signals and the composition of the microbiome. Increased activity of cDC1, cDC2 and T cells lead to both keratinocyte hyperplasia via the actions of IL-12 and IL-23, as well as a T _h_17 predominant immune response. Alterations of antimicrobial peptides (AMP’s) also occur throughout the epidermis. IHC staining localize Langerhan cells and activated dendritic cells to the epidermis and the dermo-epidermal junction. A population of epidermotropic CD8 T cells are also present. IHC staining indicates a mixed inflammatory infiltrate in the dermis, with contributions from Dendritic cells, B cells, T cells and plasma cells. Within sinus tracts, adhesion molecules are preserved, but inflammation in associated with non-keratinised sinus tracts leads to a loss of K19. The development of scarring and sinus tracts is associated with MMP2, ICAM-1 and TGF-Beta, with possible augmentation of ICAM-1 and TGF-B signaling via specific components of the microbiome. TNF-a, PGE2 and CXCL2 then lead to additional feed forward mechanisms perpetuating the inflammatory cycle.

IHC staining, in particular co-staining with cellular markers and cytokines has enabled the localization of inflammatory mediators in order to ascertain the functional aspects of infiltrating inflammatory cells in HS, particularly highlighting the strong T _h_17 polarity of inflammation in HS ^3^. A schematic representation of the pathogenesis of HS based upon the findings of this review is presented in Figure 2. This highlights the inter-relationship between inflammation and hyperkeratinization. Localization of TNF-α ^13^, IL-12 ^32^, IL-23 ^32^ and IL-32 ^4^, TLR2 ^40^, MMP2 ^13^, MMP8 ^43^ and LL-37/cathelicidin ^14, 29^ production to infiltrating dermal macrophages and lymphocytes as well as localization of IL-36 subtypes ^5^, LL-37/cathelicidin ^14, 29^, IL-1β ^32^ and IL-22 ^32^ to keratinocytes illustrate the feed forward mechanisms similar to those seen in psoriasis ^9^ and atopic dermatitis ^10^ which likely contribute to persistent inflammation in HS. Rather than keratinocytes being innocent bystanders, these IHC findings demonstrate the central role keratinocytes play as producers of key inflammatory mediators as well as mediators of products (such as TGF-β and ICAM) ^41^ that may contribute to fibroblast dysregulation and hypertrophic scarring ^46^. A remaining unanswered question includes the temporal relationship between keratinocyte hyperproliferation and the activation of inflammatory cells infiltrating the dermis and epidermis in HS.

### Insights into pathophysiology of HS

The current pathophysiological paradigm of HS is one of follicular infundibular occlusion leading to follicle rupture and a resultant inflammatory cascade ^1^. This paradigm was based on the pivotal work of Shelley and Cahn in 1955 ^47^, whom demonstrated the induction of HS after application of belladonna impregnated tape to manually epilated axillae of 12 men. Only 3 of the 12 men developed the lesions described, and infection from the manual epilation procedure could not be excluded as a cause of the lesions, but this study enabled the paradigm to slowly shift away from one of apocrinitis, which had been in place since the original descriptions of the disease ^7^. Detailed descriptions of infundibular hyperkeratosis (also termed poral occlusion) were made by Jemec *et al*. ^7^ and demonstrated the secondary involvement of apocrinitis in HS lesions. Jemec noted that poral occlusion was seen to occur alongside inflammation, but there was no suggestion of causation in one direction or another ^7^.

Although individual cases of epidermal hyperkeratosis in the absence of inflammation are noted ^7, 33^, these cases are established or chronic lesions associated with significant fibrosis which is documented to be associated with reduce inflammatory infiltrate ^7, 33^. A consistent finding in all studies of this review is the co-localization of infundibular ORS keratinocyte hyperplasia with CD3, CD4, CD8 and CD68 positive inflammatory cells expressing TNF-α, IL-12, IL-23 and IL-32 ^4, 13, 32, 40, 43^. K19 is also documented as positive in the infundibulum suggesting keratinocyte hyperplasia ^36– 38^. However, it remains unclear whether keratinocyte hyperplasia induces the inflammatory cascade or if the inflammatory cascade induces the keratinocyte hyperplasia. The presence of inflammation in clinically normal, peri-lesional HS skin is well documented ^4, 30, 33^ implying the existence of a pre-clinical inflammation preceding symptoms of follicular occlusion. This is consistent with recent findings in acne pathogenesis that suggest that inflammation precede follicular hyperkeratosis and development of microcomedones ^48^ and is also pivotal in the ongoing development of nodulocystic acne and acne scars ^49^. This pre-clinical inflammation is also consistent with the pathogenic paradigm in psoriasis and atopic dermatitis ^9, 10^ with inflammation driving epidermal hyperkeratosis and alterations in keratinocyte maturation, consistent with the spongiform infundibulfolliculitis seen in established lesions of HS ^50^. Our disparate findings in K19 staining in deep non-keratinized sinus tract epithelia with and without inflammation ^37, 38^ also fit with this paradigm. In contrast, findings which would hold consistency with the current follicular occlusion paradigm would include infundibular occlusion preceding the development of inflammation, as well as alterations to desmosomal and hemidesmosomal proteins which would allow for rupture of the occluded follicles in order to drive the development of dermal inflammation and sinus tract formation. Although Danby *et al*. ^51^ reports reduced PAS positivity in the basement membrane zone at the sebo-follicular junction associated with inflammation in HS, it is likely that the reduced basement membrane integrity is secondary to inflammation and release of TGF-β and MMP2 ^52^ (cytokines known to be altered in HS lesional skin and consistent with an abnormal wound healing response) rather than the follicular rupture being the primary driver of inflammation.

A more consistent hypothesis which accounts for the observed results of this review would be that of subclinical inflammation (due to a variety of triggers and immunological primers as illustrated in Figure 2) driving keratinocyte proliferation in the interfollicular epidermis and the follicular ORS, with follicular occlusion being a secondary phenomenon (mediated by TLR2 and IL-1α as documented in the development of comedones) ^53^. The development of sinus tracts and hypertrophic scarring may also be mediated by the keratinocyte inflammatory response given the alterations in important wound healing mediators including TGF-β, ICAM-1 and comparisons by other authors of an altered wound healing response ^8^ in HS. This comparison would be appropriate given the high levels of dermal MMP2 ^13^ and MMP8 ^43^; the loss of keratinocyte maturation markers (K2e, K10, K19) ^36– 38^ adhesion molecules (DG1 and DCN2) ^38^ in the non keratinized inflamed epithelium of the deep dermis; suppressed levels of ICAM-1 ^41^ (seen impaired wound healing ^54^) and TGF-β ^41^ which leads to the dysregulation of TGF- β receptor ratio on fibroblasts which is linked with the development of hypertrophic scarring ^46, 54^ seen in HS. These alterations to keratinocyte maturation are reminiscent of epithelial mesenchymal transition (EMT) ^52^ which may also explain the presence of free keratinocytes in the dermis in established lesions of HS ^7, 36^. Indeed, as ICAM-1 is up-regulated by pro-inflammatory mediators ^54^, the low level of ICAM-1 noted appears paradoxical, however specific bacteria (including *Porphyromonas* species) which have been associated with HS ^44, 55^ can suppress ICAM-1 production as an immune evasion strategy ^56^. This implies that exogenous triggers (possibly including bacterial stimuli) can be a common cause for the initial inflammatory cascade as well as the development of tunneling and hypertrophic scarring in HS.

## Conclusions

This systematic review of immunohistochemical staining of lesions in HS has highlighted the heterogeneity of studies and the methodological issues, which bring into question some of the results of IHC staining in HS lesions. The design of studies and variable reporting of potential confounding factors (such as ongoing or previous treatments) makes it impossible to compare staining intensity across studies. The results of existing studies suggest a florid inflammatory reaction comprising of T-lymphocytes, macrophages and dendritic cells with a strong Th-17 signature along with a keratinocyte mediated IL-36 inflammatory loop associated with keratinocyte hyperproliferation. The follicular occlusion paradigm as a primary driver of HS is unclear given the findings of this review and other histological and cytokine studies and inflammation as a primary driver of disease with secondary hyperkeratosis and occlusion is a plausible hypothesis.

## Data availability

All data underlying the results are available as part of the article and no additional source data are required

### Extended data

OSF: Extended data. Data collection sheet. https://doi.org/10.17605/OSF.IO/2JKPW ^27^

Licence: CC0 1.0 Universal

### Reporting guidelines

OSF: PRISMA Checklist for ‘A systematic review and critical evaluation of immunohistochemical associations in hidradenitis suppurativa’. https://doi.org/10.17605/OSF.IO/2JKPW ^27^

Licence: CC0 1.0 Universal
