# Supplementary material for: A systematic review and critical evaluation of immunohistochemical associations in hidradenitis suppurativa
Source: F1000Res. 2019 Jun 17;7:1923. Originally published 2018 Dec 11. [Version 2] doi: 10.12688/f1000research.17268.2 (PMC6593329; doi:10.12688/f1000research.17268.2)
Supplement: Peer review history

# Peer review: Reviewer response for version 2

**DOI:** 10.5256/f1000research.21501.r49996

**Published:** 2019-06-25

**Authors:** Martin M. Okun

**Affiliations:** 1 Fort HealthCare, Fort Atkinson, WI, USA

My concerns have been satisfactorily addressed with these changes. I support indexing.

I confirm that I have read this submission and believe that I have an appropriate level of expertise to confirm that it is of an acceptable scientific standard.

# Peer review: Reviewer response for version 1

**DOI:** 10.5256/f1000research.18880.r45666

**Published:** 2019-03-19

**Authors:** Martin M. Okun

**Affiliations:** 1 Fort HealthCare, Fort Atkinson, WI, USA

This is a thorough, thoughtful, and valuable contribution to the scientific literature on pathogenesis of hidradenitis suppurativa.

 The principal advances of the systematic review are:

- cogently advances a reasonable hypothesis to explain decreased levels of inflammatory marker density in some studies as due to the lack of treatment interruption;
- links the presence of certain hyperproliferative keratin markers (K19) to the presence of concomitant inflammation
- collates evidence from multiple studies demonstrating the presence of keratinocyte-derived pro-inflammatory biomarkers, reinforcing the concept that keratinocytes are actively contributing to the inflammatory milieu

The authors advance the hypothesis that follicular occlusion is secondary to inflammation, based on:

- absence of evidence of follicular occlusion without inflammation (though absence of evidence is not equivalent to the evidence of absence)
- presence of inflammation in clinically normal perilesional skin (though inflammation could be spill-over from adjacent inflamed skin)
- analogies with other inflammatory skin diseases
- presence of K19 staining only in inflamed sinus tracts (though the relevance of this observation for the pathogenesis of HS is uncertain)

In short, this hypothesis is plausible but the conclusion that "primary follicular occlusion as a pathogenic paradigm and the principal driver of HS is not consistent with the findings of this review" seems too sweeping a statement based on the information provided. The authors should consider altering this conclusion in line with the limitations of available data.

 As a minor issue, there is an unnecessary repetition in the second sentence of the Immunohistochemistry results, epidermis section:  "Increased K6, K16 and K17 staining were increased..."

I confirm that I have read this submission and believe that I have an appropriate level of expertise to confirm that it is of an acceptable scientific standard, however I have significant reservations, as outlined above.

# Peer review: Reviewer response for version 1

**DOI:** 10.5256/f1000research.18880.r42315

**Published:** 2019-02-25

**Authors:** Gregor B. E. Jemec

**Affiliations:** 1 University of Copenhagen, Copenhagen, Denmark

The authors provide a systematic review of immunohistochemical studies of hidradenitis suppurativa (HS).

 The clearly stated objectives are:

1. To collate and describe all published reports of immunohistochemical studies in HS.
2. To critically evaluate the sampling, laboratory and analysis techniques used in each study to determine if comparisons can be made across studies.

The review was registered with PROSPERO and conducted in line with the PRISMA. The STROBE statement was used to assess the observational studies included in the study. A PRISMA flow chart and a search strategy are provided accordingly.

 The authors adequately discuss the confounding factors and risk of bias, which both are significant weaknesses identified in the literature by this manuscript based on limited studies. 

 Only 22 articles were identified describing results from 494 HS patients (average 22 pts/study) and only 168 controls. Furthermore, 87 unique immunohistochemical targets were identified adding to the scarcity of hard data. It is therefore less surprising that conflicting data were found. The authors are however able to provide a realistic analysis of the data taking these limitations into account, and, in addition, provide coherent analyses and a testable paradigm for the pathomechanisms of HS.

 The paper provides an excellent overview of the limited number of explorative immunohistochemical studies of HS, and thus provides an important stepping-stone to further studies.

I confirm that I have read this submission and believe that I have an appropriate level of expertise to confirm that it is of an acceptable scientific standard.
